# Design and Development of D‒α‒Tocopheryl Polyethylene Glycol Succinate‒*block*‒Poly(ε-Caprolactone) (TPGS−*b*−PCL) Nanocarriers for Solubilization and Controlled Release of Paclitaxel

**DOI:** 10.3390/molecules26092690

**Published:** 2021-05-04

**Authors:** Osman Yusuf, Raisuddin Ali, Abdullah H. Alomrani, Aws Alshamsan, Abdullah K. Alshememry, Abdulaziz M. Almalik, Afsaneh Lavasanifar, Ziyad Binkhathlan

**Affiliations:** 1 Department of Pharmaceutics, College of Pharmacy, King Saud University, P.O. Box 2457, Riyadh 11451, Saudi Arabia; osmohamed@ksu.edu.sa (O.Y.); ramohammad@ksu.edu.sa (R.A.); aomrani@ksu.edu.sa (A.H.A.); aalshamsan@ksu.edu.sa (A.A.); aalshememry@ksu.edu.sa (A.K.A.); 2 Nanobiotechnology Research Unit, College of Pharmacy, King Saud University, P.O. Box 2457, Riyadh 11451, Saudi Arabia; 3 Life Science and Environment Research Institute, King Abdulaziz City of Science and Technology, Riyadh 11461, Saudi Arabia; aalmalik@kacst.edu.sa; 4 Faculty of Pharmacy and Pharmaceutical Sciences, University of Alberta, Edmonton, AB T6G 2H7, Canada; afsaneh@ualberta.ca; 5 Department of Chemical and Material Engineering, University of Alberta, Edmonton, AB T6G 2V4, Canada

**Keywords:** D‒α‒tocopheryl polyethylene glycol succinate, block copolymer, nanocarriers, polymeric micelles, paclitaxel

## Abstract

The objective of this study was to synthesize and characterize a set of biodegradable block copolymers based on TPGS-*block*-poly(ε-caprolactone) (TPGS-*b*-PCL) and to assess their self-assembled structures as a nanodelivery system for paclitaxel (PAX). The conjugation of PCL to TPGS was hypothesized to increase the stability and the drug solubilization characteristics of TPGS micelles. TPGS-*b*-PCL copolymer with various PCL/TPGS ratios were synthesized via ring opening bulk polymerization of ε-caprolactone using TPGS, with different molecular weights of PEG (1–5 kDa), as initiators and stannous octoate as a catalyst. The synthesized copolymers were characterized using ^1^H NMR, GPC, FTIR, XRD, and DSC. Assembly of block copolymers was achieved via the cosolvent evaporation method. The self-assembled structures were characterized for their size, polydispersity, and CMC using dynamic light scattering (DLS) technique. The results from the spectroscopic and thermal analyses confirmed the successful synthesis of the copolymers. Only copolymers that consisted of TPGS with PEG molecular weights ≥ 2000 Da were able to self-assemble and form nanocarriers of ≤200 nm in diameter. Moreover, TPGS_2000_-*b*-PCL_4000_, TPGS_3500_-*b*-PCL_7000_, and TPGS_5000_-*b*-PCL_15000_ micelles enhanced the aqueous solubility of PAX from 0.3 µg/mL up to 88.4 ug/mL in TPGS_5000_-*b*-PCL_15000_. Of the abovementioned micellar formulations, TPGS_5000_-*b*-PCL_15000_ showed the slowest in vitro release of PAX. Specifically, the PAX-loaded TPGS_5000_-*b*-PCL_15000_ micellar formulation showed less than 10% drug release within the first 12 h, and around 36% cumulative drug release within 72 h compared to 61% and 100% PAX release, respectively, from the commercially available formulation (Ebetaxel^®^) at the same time points. Our results point to a great potential for TPGS-*b*-PCL micelles to efficiently solubilize and control the release of PAX.

## 1. Introduction

D‒α‒tocopheryl polyethylene glycol succinate (TPGS) is a water-soluble polyethylene glycol (PEG) derivative of vitamin E. It is widely used in various drug delivery systems [1]. The presence of PEG in the molecule extends the circulation time of the drug in plasma, while vitamin E enhances the cellular uptake of the drug. Several derivatives of TPGS (i.e., with different PEG chain lengths) have been studied. However, TPGS_1000_ (i.e., PEG_1000_) is the most studied and widely used derivative [2]. TPGS has a hydrophilic–lipophilic balance (HLB) value of 13 and, upon addition to water at a concentration higher than its critical micelle concentration (CMC) (0.02% *w*/*w*), forms micelles with diameters in the nano range [1,3]. The US FDA has approved TPGS as a safe pharmaceutical excipient for use in drug formulation. In recent years, TPGS has been extensively used in the pharmaceutical industry. It has been used as an absorption enhancer, emulsifier, solubilizer, additive, permeation enhancer, and stabilizer. TPGS has also been used as an inhibitor of P-glycoprotein (P-gp) to enhance the oral bioavailability of P-gp substrates [2,3]. It has also been used to enhance drug uptake in the brain as well as in tumor cells [4,5].

Poly(ε-caprolactone) (PCL) is a biodegradable and biocompatible polyester that has been extensively studied for controlled drug delivery and tissue engineering applications [6,7]. It has the advantage of being compatible with a wide range of drugs, which allows homogenous drug distribution in the polymer matrix. Moreover, PCL exhibits a long degradation time leading to sustained drug release that could last for months in cases where drug release is dependent on polymer matrix degradation [8]. Compared to other core-forming blocks in the poly(ester) category, such as PLGA and PLA, PCL is more hydrophobic, which makes it more compatible with hydrophobic drugs. The hydrophobicity of PCL has pushed the CMC of PEG-*b*-PCL to extremely low concentrations (in 100 nM range) [9]. PCL-based polymeric micelles have been successfully used to deliver a variety of lipophilic drugs, including P-gp inhibitors such as Cyclosporine A and valspodar [10,11] and some chemotherapeutic agents such as methotrexate and paclitaxel [12,13,14].

Paclitaxel (PAX) is a complex diterpene with the empirical formula of C_47_H_51_NO_14_ and a molecular weight of 853.91 Da. This anticancer agent was discovered in a U.S. National Cancer Institute program in 1967. It is isolated from *Taxus brevifolia* and *Taxus yannanensis*. PAX is a biopharmaceutical classification system (BCS) class IV drug (i.e., low solubility and low permeability). It is highly lipophilic (LogP 3.66) and practically insoluble in water (0.3 μg/mL) [15,16]. However, PAX is soluble in DMSO, acetonitrile, methanol, and ethanol. It is also soluble in a mixture of 50% Cremophor^®^ EL (currently known as Kolliphor^®^ EL) and 50% anhydrous ethanol, which is the formula currently used in clinical settings (Taxol^®^). According to the current chemotherapy protocols that involve Taxol^®^ administration, all patients must be pretreated with corticosteroids (e.g., dexamethasone), H_1_ antagonists (e.g., diphenhydramine), and H_2_ antagonists (e.g., ranitidine) prior to Taxol^®^ administration in order to prevent severe hypersensitivity reactions. In fact, the hypersensitivity reactions reported in association with the use of Taxol^®^ were found to be due to the presence of Kolliphor^®^ EL [17,18,19].

There are currently two marketed products for PAX that offer the advantage of being Kolliphor-EL-free formulations, namely albumin-bound PAX (Abraxane^®^) and a methoxy PEO-*b*-PDLLA micellar formulation (Genexol^®^-PM) [20]. Although these formulations serve as safer alternatives for Taxol^®^, their clinical pharmacokinetic profiles suggest that they likely act more as solubilizers of PAX than as stable drug carriers [20,21]. Therefore, there is still a need to develop a nanodelivery system for PAX that can control the release of the drug and prolong its circulation time in the blood so that it can be used as a tumor-targeted delivery system. Indeed, extensive research work has resulted in the development of several nanodelivery systems for PAX that are currently at different stages of preclinical and clinical development, including liposomes, polymeric micelles, and polymeric nanoparticles [20]. 

Bernabeu et al. reported on a nanoformulation of PAX based on TPGS_1000_-*b*-PCL_30000_ nanoparticles [22]. The nanoparticles showed a sustained in vitro release of PAX with only 20% and 45% cumulative drug release at 24 and 96 h, respectively. Moreover, the in vitro cytotoxicity (48 h incubation) of PAX-loaded TPGS_1000_-*b*-PCL_30000_ nanoparticles was studied using two human breast cancer cell lines (MCF-7 and MDA-MB-231) [22]. The results showed that PAX-loaded TPGS_1000_-*b*-PCL_30000_ nanoparticles had a significantly higher in vitro cytotoxicity in both cell lines compared to PAX in solution and in the commercially available formulation (Abraxane^®^) [22]. Specifically, in the case of MDA-MB-231, the IC_50_ value for PAX-loaded TPGS_1000_-*b*-PCL_30000_ nanoparticles was 7.8 times lower than Abraxane^®^. Furthermore, following administration of a 6 mg/kg single intravenous dose to rats, PAX-loaded TPGS_1000_-*b*-PCL_30000_ showed a significantly lower plasma clearance of PAX compared to Taxol^®^ and Abraxane^®^. This resulted in a 2.7- and 3.7-fold increase in plasma AUC of PAX in TPGS_1000_-*b*-PCL_30000_ compared to Taxol^®^ and Abraxane^®^, respectively [22]. In another study, Bernabeu et al. showed that TPGS_1000_-*b*-PCL_10500_ nanoparticles had higher cellular uptake (6 h incubation) and in vitro cytotoxicity (48 and 72 h incubation) compared to methoxy PEO_5000_-*b*-PCL_9500_ nanoparticles in both cell lines (i.e., MCF-7 and MDA-MB-231) [23]. 

Here, we synthesized and characterized biodegradable block copolymers of TPGS-*b*-PCL using different TPGS analogues (different molecular weights of PEG) and different molecular weights of PCL. TPGS-*b*-PCL copolymers are expected to form nanocarriers capable of solubilization and delivery of hydrophobic drugs. The presence of TPGS as the shell-forming block can not only support better solubilization of hydrophobic cargos, but also enhance stability and delivery of P-gp substrates (including PAX) to P-gp-overexpressing cells [1,23,24,25]. In this paper, the potential of TPGS-*b*-PCL nanocarriers for solubilization and controlled release of PAX was explored.

## 2. Materials and Methods

### 2.1. Materials

D-α-Tocopherol polyethylene glycol 1000 succinate (TPGS_1000_), ε-Caprolactone (97%), and stannous octoate were purchased from Sigma-Aldrich (St. Louis, MO, USA). D-α-Tocopherol polyethylene glycol 2000 succinate (TPGS_2000_), D-α-Tocopherol polyethylene glycol 3500 succinate (TPGS_3500_), and D-α-Tocopherol polyethylene glycol 5000 succinate (TPGS_5000_) were obtained from Wuhan Jason Biotech Co., Ltd. (Wuhan, Hubei, China). Paclitaxel (99%) was purchased from Shaanxi Sciphar Biotechnology Co., Ltd. (Xianyang, Shijiyiuan, China). Ebetaxel^®^ (300 mg/50 mL, concentrate for infusion; Ebewe Pharma, Unterach, Austria; Lot# HN5538), a generic of Taxol^®^ approved by the US FDA, was obtained from King Khalid University Hospital Pharmacy (Riyadh, Saudi Arabia). Deuterated chloroform (CDCl_3_, 99.8%) was purchased from Cambridge Isotope Laboratories Inc. (Tewksbury, MA, USA). Polysorbate 80 (Tween 80) was purchased from Bio Basic Inc. (Markham, ON, Canada). Potassium dihydrogen orthophosphate, dipotassium hydrogen orthophosphate, and potassium chloride were obtained from BDH Chemical Ltd. (Poole, England). Acetonitrile, methanol, and water were all HPLC grade and were purchased from BDH Chemical Ltd. (Poole, England). All other chemicals were reagent grade. 

### 2.2. Methods

#### 2.2.1. Synthesis of TPGS-b-PCL Copolymers 

TPGS-*b*-PCL copolymers with various PCL/TPGS ratios were synthesized by ring-opening bulk polymerization of ε-caprolactone using TPGS as an initiator and stannous octoate as a catalyst [11,26,27]. TPGS (with PEG molecular weights of 1, 2, 3.5, or 5 kDa), ε-caprolactone, and stannous octoate (0.002 eq of monomer) were added to a previously flamed 10 mL ampoule, nitrogen purged, then sealed under vacuum. The polymerization reactions were allowed to proceed for several hours (4–5 h) at 140 °C in oven. The reaction was terminated by cooling the product to room temperature.

#### 2.2.2. Characterization of TPGS-b-PCL Copolymers 

##### Molecular Weight and Polydispersity of TPGS-b-PCL Copolymers

^1^H NMR analyses were performed on a Bruker Ultra shield 500.133 MHz spectrometer using CDCl_3_ as a solvent. TPGS-*b*-PCL copolymers were dissolved in CDCl_3_ at a concentration range of 10−15 mg/mL. ^1^H NMR spectra were generated using tetramethylsilane as an internal standard. Baseline correction, calibration, and processing were performed using Topspin software. The degree of polymerization of ε-CL in each copolymer was calculated from the ^1^H NMR spectra by examining the peak intensity ratio of methylene protons of PCL segment (–O–CH_2_) and the methylene protons of PEG segment (–O–CH_2_–CH_2_) at δ =4.07 ppm and 3.65 ppm, respectively. 

The number-averaged molecular weights, weight-averaged molecular weights, and molecular weight distributions of the synthesized copolymers were determined by gel permeation chromatography (GPC) (Viscotek TDA 305-040 Triple Detector Array, Viscotek Corp., Houston, TX, USA). Samples (100 µL from 15 mg/mL polymer stock solutions in THF) were injected into an 8.0 × 300 mm Viscotek T6000M column (Viscotek Corp., Houston, TX, USA) with a guard column. The mobile phase (THF) was delivered at a flow rate of 1 mL/min. The calibration curve was established using polystyrene standards (molecular weight range: 1570–46,500).

##### Fourier Transform Infrared (FTIR) Spectroscopy

The FTIR spectra of the synthesized copolymers were obtained using an FTIR spectrophotometer (PerkinElmer, USA). Copolymer samples were ground with potassium bromide (spectroscopic grade) and compressed into a thin disk using a hydraulic press before scanning from 4400 to 400 cm^−1^.

##### X-ray Diffraction (XRD)

An X-ray diffractometer was used to study the crystalline states of the synthesized copolymers. Samples of the copolymers and TPGS were loaded into the XRD instrument (automated Rigaku Ultima IV). The X-ray diffractogram of the investigated sample was collected using 2theta (2θ) scan axis mode, with the scan speed set at 0.5°/min and a covering scan range of 3.0–50.0°. The scanning process was performed at room temperature.

##### Differential Scanning Calorimetry (DSC)

Thermograms of TPGS and TPGS-*b*-PCL were obtained using DSC (DSC-60, Shimadzu, Japan). Samples (3–5 mg) were loaded in an aluminum pan and sealed with aluminum lids using a crimper. The samples were then thermally scanned against an empty aluminum pan with lid at a heating rate of 10° C/min, covering a temperature range of 25–200 °C. Nitrogen purging at 40 mL/min was used during scanning. The TA-60WS thermal analysis software was used to calculate the thermal parameters of the scanned sample.

#### 2.2.3. Preparation of Drug-Free and PAX-Loaded TPGS-b-PCL Micelles

Assembly of block copolymers was achieved via the cosolvent evaporation method. Briefly, drug and TPGS-*b*-PCL copolymer were dissolved in a water-miscible organic solvent and added in a dropwise manner (1 drop/15 s) to distilled water with stirring. The remaining organic solvent was removed by evaporation at room temperature overnight under vacuum. The prepared micelles were purified by centrifugation (at 13,000 rpm for 5 min) to remove any of the nonentrapped drug and polymer aggregates. Generally, micelles were freshly prepared prior to each experiment. In instances where micelles needed to be stored for further use, they were kept at 4 °C. 

#### 2.2.4. Characterization of TPGS-b-PCL Micelles

##### Size, Polydispersity, and CMC

Mean diameter, polydispersity, and CMC of self-assembled structures in aqueous media were defined by dynamic light scattering (DLS) (Zetasizer™ Nano ZS, Malvern Instrument Ltd., Malvern, UK).

To determine CMC, DLS measurements were carried out as previously described [28]. A serial dilution from the micellar solution was prepared from each polymer and the intensity of scattered light was recorded in triplicate for each concentration point. The CMC was calculated from the abrupt increase in the intensity of scattered light (intersection point).

##### Morphology

Morphology of the assembled structures in the present study was characterized by transmission electron microscopy (TEM). An aqueous droplet of micellar solution (20 μL) was placed on a copper-coated grid (Ted Pella, Inc., Redding, CA, USA). The grid was held horizontally for 20 s to allow the colloidal aggregates to settle. A drop of 2% solution of phosphotungstic acid (PTA) in PBS (pH = 7.0) was then added to provide the negative stain. After 1 min, the excess fluid was removed using a strip of filter paper. The samples were then allowed to dry at room temperature and loaded into a JEOL JEM-1400 transmission electron microscope (JAPAN) operating at an acceleration voltage of 120 kV. Images were recorded with a Gatan Ultrascan high-resolution digital camera and processed with JADAS (the JEOL automated data acquisition system) software.

##### Encapsulation Efficiency and Drug Loading

PAX level in the supernatant was determined using a previously published HPLC assay method with minor modification [29]. The HPLC system consisted of a Waters Model 1515 HPLC pump, Waters Autosampler Model 717 plus (Waters Inc., Bedford, MA, USA), and Waters 2487 dual absorbance UV detector (Waters Inc., Bedford, MA, USA) governed by a computer running Empower software (version 1154). Quantification of PAX was achieved via an isocratic elution using acetonitrile and water (70:30) as mobile phase delivered at a flow rate of 1 mL/min (at ambient temperature) through a C_18_ analytical column (Sunfire^®^; 250 mm length × 4. 6 mm i.d., 5 µm particle size). The UV detection was performed at a wavelength of 227 nm and the injection volume was 30 µL. Drug loading content and encapsulation efficiency were determined using the following equations:

(1) $$\mathrm{PAX}\;\mathrm{loading}\;\left(\%\;w/w\right)=\frac{\mathrm{Amount}\;\mathrm{of}\;\mathrm{loaded}\;\mathrm{PAX}\;\left(\mathrm{mg}\right)}{\mathrm{Amount}\;\mathrm{of}\;\mathrm{polymer}\;\left(\mathrm{mg}\right)+\;\mathrm{Amount}\;\mathrm{of}\;\mathrm{loaded}\;\mathrm{PAX}\;\left(\mathrm{mg}\right)}\times 100$$

(2) $$\mathrm{Encapsulation}\;\mathrm{efficiency}\;\left(\%\right)=\frac{\mathrm{Amount}\;\mathrm{of}\;\mathrm{loaded}\;\mathrm{PAX}\;\left(\mathrm{mg}\right)}{\mathrm{Amount}\;\mathrm{of}\;\mathrm{PAX}\;\mathrm{added}\;\left(\mathrm{mg}\right)}\times 100$$

#### 2.2.5. In Vitro Drug Release

Release of paclitaxel from the nanocarriers was assessed in 50 mL phosphate-buffered saline (pH 7.4) containing 0.5% *w*/*v* polysorbate 80 at 37 °C (to provide sink condition), as previously described [22,23,30,31,32]. In this method, volumes of polymeric micelles equivalent to 50 μg of PAX were placed into a dialysis membrane bag (molecular weight cut off 12–14 kDa). The samples were placed in a water bath and shaken at 100 rpm 37 °C. Samples (in triplicate) of 1 mL were taken from the release medium at predetermined time intervals and replaced by fresh medium. The release profile of the micellar formulation with highest loading of paclitaxel was compared to the release profiles of free drug (PAX dissolved in ethanol) and Ebetaxel^®^ (the commercially available formulation). 

The PAX release profiles from the ethanolic solution, Ebetaxel^®^, and the polymeric micellar formulations were compared using a model independent approach (calculation of difference factor and similarity factor) [33]. The difference factor (*f*_1_), which is the percent error between the two curves over all time points, was calculated as follows:

(3) $$f_{1}=\frac{\sum_{j=1}^{n}\left|R_{j}-T_{j}\right|}{\sum_{j=1}^{n}Rj}\times 100$$

where *n* is the sampling number, and *R_j_* and *T_j_* are the percent PAX released from reference formulation and test, respectively, at time *j*. The similarity factor (*f*_2_) was calculated as follows:

(4) $$f_{2}=50\times log\left\{{\left[1+\left(1/n\right)\overset{n}{\underset{j=1}{\sum }}{\left(R_{j}-T_{j}\right)}^{2}\right]}^{-\frac{1}{2}}\times 100\right\}$$

In general, *f*_1_ values lower than 15 (0–15) and *f*_2_ values higher than 50 (50–100) indicate similar release profile [34].

#### 2.2.6. Data Analysis

Statistical analysis was performed either using paired Student’s *t*-test or one-way ANOVA with post hoc analysis using the Tukey–Kramer multiple comparison test. The significance level (α) was set at 0.05. All experiments were conducted in triplicate unless stated otherwise in the text, tables, or figures. The results were represented as mean ± standard deviation (SD).

## 3. Results and Discussion

### 3.1. Synthesis and Characterization of TPGS-b-PCL Copolymers 

Although a few reports on TPGS-*b*-PCL copolymers have been published recently, only few molecular weights have been studied [22,23,35,36]. Moreover, in all these previous studies, TPGS_1000_ (PEG MW = 1000) was the only form used, and TPGS-*b*-PCL copolymers have always been prepared as nanoparticles (i.e., not polymeric micelles). Here, we tried to extensively study the influence of the molecular weight of both PEG (in TPGS) and PCL on the physicochemical properties of the synthesized copolymers as well as on their self-assembled structures. We also tried to develop PAX-loaded micelles and report on their characteristics including size, polydispersity, drug encapsulation efficiency, and in vitro drug release profile. For this, TPGS with different molecular weights of PEG (1–5 kDa) were used to synthesize TPGS-*b*-PCL copolymers with different molecular weights of PCL. All the copolymers were successfully synthesized and characterized using different analytical techniques. Table 1 shows the different TPGS-*b*-PCL copolymers synthesized.

#### 3.1.1. ^1^H NMR

**Figure 1:**
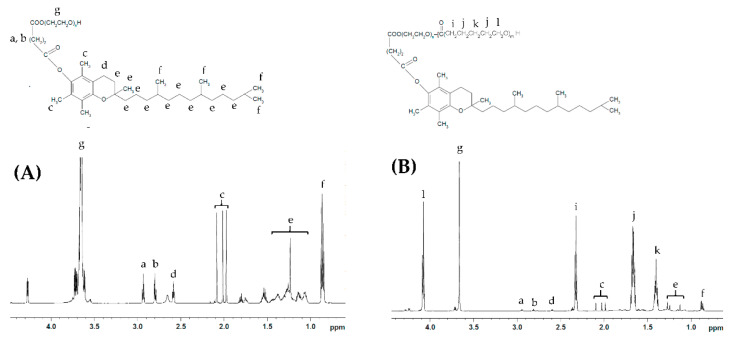
Representative ^1^H NMR spectra of TPGS_1000_ (**A**) and TPGS_1000_-*b*-PCL_4000_ (**B**).

Representative NMR spectra of TPGS and the synthesized copolymers are shown in Figure 1 and Figure S1. TPGS-*b*-PCL copolymers show the following signals: δ =4.07, 2.32, 1.67 and 1.38 ppm, which were assigned to (H–O–C**H_2_**–), (–CO–C**H_2_**–), (–CO–CH_2_–C**H_2_**–CH_2_–C**H_2_**–CH_2_–O–H), and (–CO–CH_2_–CH_2_–C**H_2_**–CH_2_–CH_2_–O–H) of the PCL segment, respectively. The peak at δ =3.65 ppm was assigned to the methylene protons of the PEG unit in the TPGS segment ‒COO–(C**H_2_**–C**H_2_**‒O–)_n_. The peaks, including those in the aliphatic region (δ = 0.87–1.28 ppm) as well as those at 2.59–2.95 ppm, correspond to various protons of the tocopheryl succinate moiety (Figure 1A). The molecular weights and the compositions of the synthesized copolymers were determined by ^1^H NMR based on the intensity ratio between the peaks at δ = 4.07 ppm and 3.65 ppm. ^1^H NMR data were found to be consistent with the theoretical values and revealed a well-defined composition of the copolymers. The characteristics of the synthesized TPGS-*b*-PCL copolymers are summarized in Table 1. 

#### 3.1.2. GPC

**Figure 2:**
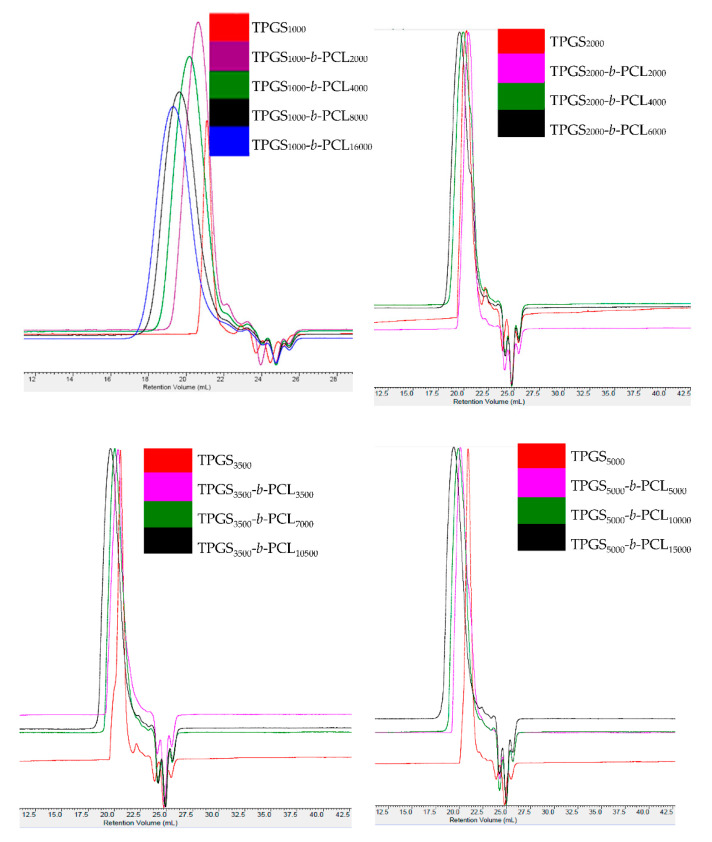
GPC chromatograms of TPGS_1000_, TPGS_2000_, TPGS_3500_, TPGS_5000_, and their corresponding PCL copolymers.

GPC analysis further confirmed the successful polymerization with a Đ range from 1.05–1.9. Figure 2 and Figure S2 show the GPC chromatograms of TPGS and the synthesized TPGS-*b*-PCL copolymers. As expected, compared to TPGS, the peaks for all synthesized TPGS-*b*-PCL copolymers shifted to lower retention volumes (higher molecular weights). For example, while TPGS_5000_ had a retention volume of 21 mL, TPGS-*b*-PCL copolymers had retention volumes of 20.1, 19.9, and 19.4 mL for TPGS_5000_-b-PCL_5000_, TPGS_5000_-b-PCL_10000_, and TPGS_5000_-b-PCL1_5000_, respectively (Figure 2). The relative number-average molecular weights determined by the GPC were in good agreement with those obtained from ^1^H NMR (Table 1). Each copolymer eluted as a single peak with no TPGS peak and no shoulder peak, confirming that the polymerization reaction was successful. It was noticed that Đ slightly increased as the molecular weight of the TPGS increased. Đ average values of 1.16, 1.56, 1.57 and 1.67 were calculated for TPGS_1000_-, TPGS_2000_-, TPGS_3500_-, and TPGS_5000_-based copolymers, respectively. 

#### 3.1.3. FTIR

**Figure 3:**
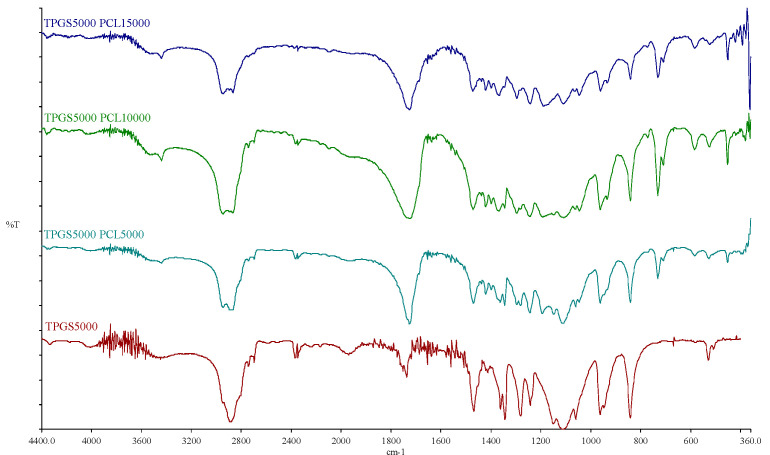
FTIR spectra of TPGS_5000_, TPGS_5000_-*b*-PCL_5000_, TPGS_5000_-*b*-PCL_10000_, and TPGS_5000_-*b*-PCL_15000_ copolymers.

The FTIR spectra of TPGS_1000_, TPGS_2000_, TPGS_3500_, TPGS_5000_, and their corresponding copolymers are shown in Figure S3 and Figure 3. For the TPGS moieties, the characteristic bands for hydroxyl and carbonyl groups appeared at 3452–3454 cm^−1^ and 1737–1738 cm^−1^, respectively. For the synthesized copolymers, the carbonyl bands were shifted to 1724–1726 cm^−1^ with stronger intensity, due to the formation of PCL. Moreover, it was noticed that with the increasing molecular weight of PCL, the band at 2944–2945 cm^−1^ increased; this is the characteristic aliphatic CH stretching band of ε-CL, as previously reported [37,38]. On the other hand, the absorption band of CH stretching vibration in PEO and α-tocopheryl succinate moieties at 2884–2886 cm^−1^ decreased while the hydroxyl band was shifted toward 3438–3448 cm^−1^. Similar observations were reported by Zhang et al. [36] when the FTIR spectrum of TPGS was compared to TPGS_1000_-*b*-PCL_17000_ (Mn ~ 18,500 Da). Moreover, Shin et al. also reported similar shifts in the abovementioned bands when the FTIR spectrum of methoxy PEG was compared to the spectra of methoxy PEO_5000_-*b*-PCL with different molecular weights of PCL (Mn ~ 4000–17,000 Da) [38]. These observations verify the presence of intermolecular interactions occurring during the polymer synthesis [36,39].

#### 3.1.4. XRD 

**Figure 4:**
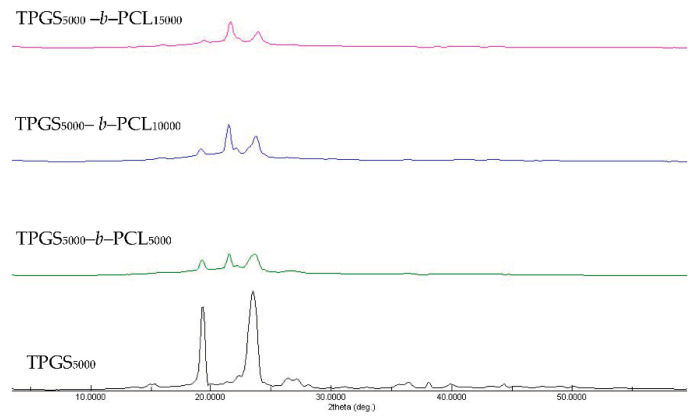
XRD diffractograms of TPGS_5000_, TPGS_5000_-*b*-PCL_5000_, TPGS_5000_-*b*-PCL_10000_, and TPGS_5000_-*b*-PCL_15000_ copolymers.

The X-ray diffractograms of TPGS and the synthesized copolymers are shown in Figure S6 and Figure 4. The diffraction pattern of neat TPGS exhibited good crystallinity with two sharp peaks appearing at 2θ = 19.3° and 23.5° [39]. The synthesized copolymers showed different crystallinity behaviors according to their PCL content. In addition to TPGS peaks, the copolymers with a low molecular weight of PCL showed strong peaks at 2θ = 21.5° and 23.8°, corresponding to the reported PCL crystalline units [40,41,42]. However, TPGS peaks tended to decrease with increasing the molecular weight of PCL, in a manner similar to that reported previously with PEO-*b*-PCL copolymers [38,40,42]. 

Sun et al. used temperature-dependent XRD to study the crystallization process of a series of PEO-*b*-PCL copolymers compared to PEO and PCL homopolymers with asymmetric block compositions [42]. For PEO-*b*-PCL, microphase separation was observed and explained by the tendency of PCL and PEO blocks to crystallize separately. More importantly, the relative block length determined which block crystallized first. The temperature-dependent XRD measurements revealed that when the length of the PEO block was longer it crystallized first, leading to imperfect crystallization of the PCL block and vice versa. For instance, during the cooling process of PEO_5000_-*b*-PCL_9200_, the characteristic diffraction peak for the PCL crystals (2θ = 21.4) emerged first at 65 °C, whereas that of the PEO crystals (2θ = 19.3) appeared later at 50 °C [42]. This finding means that PCL crystals formed (nucleated) first and prevented further growth of PEO crystals, which was reflected in the higher relative intensity of the characteristic diffraction peak of PCL compared to the PEO characteristic peak. Indeed, this is in line with our findings here (Figure S6 and Figure 4). We found that the higher the molecular weight of PCL, the higher the diffraction peak intensity at 2θ = 21.6 compared to 2θ = 19.3 (characteristic diffraction peak of PEO part of TPGS). 

#### 3.1.5. DSC

**Figure 5:**
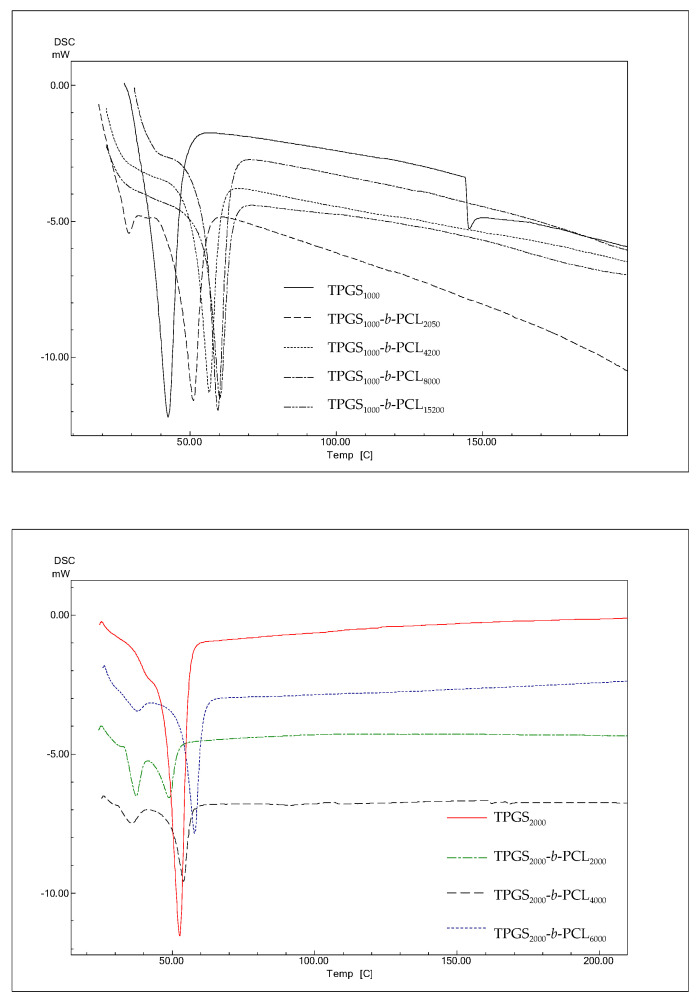

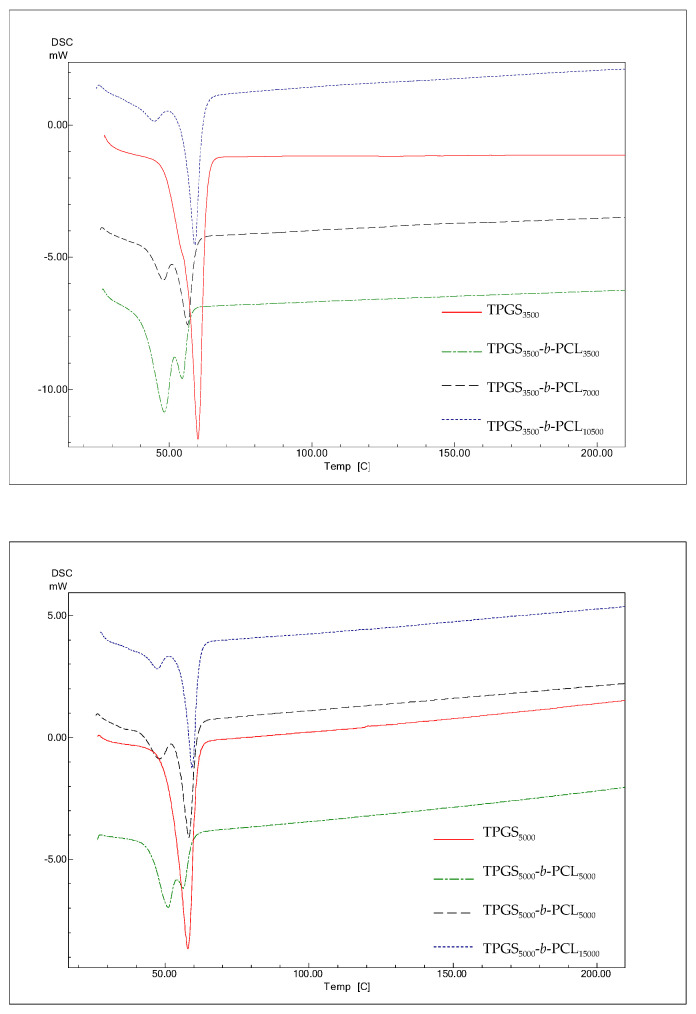
DSC thermograms of TPGS_1000_, TPGS_2000_, TPGS_3500_, TPGS_5000_, and their corresponding PCL copolymers.

The thermal properties of the different TPGS and TPGS-*b*-PCL copolymers were investigated using DSC. According to previous reports, PCL homopolymers exhibit sharp endothermic peak at 59–64 °C depending on the size of crystals and the molecular weight of the polymer [42,43,44]. As shown in Figure 5, DSC thermograms of TPGS_1000_, TPGS_2000_, TPGS_3500_, and TPGS_5000_ displayed strong peaks at 42.4 °C [39], 54.3 °C, 58.7 °C, and 56.7 °C, respectively corresponding to the melting points of PEG at different molecular weights. This was in line with what has been previously reported, i.e., as PEG length increases, its melting temperature increases [45,46]. The results also revealed that the synthesized copolymers showed one endothermic peak located in between the reported melting transitions of PCL homopolymer [39] and their precursor TPGS. The melting temperatures of the copolymers correlated well with the PCL content. The melting temperatures of the copolymers depend on the degree of crystallinity, which increases as the PCL ratio increases [38,39,47]. 

Suksiriworapong et al. investigated the effects of different hydrophobic chain lengths of TPGS_1000_-*b*-PCL copolymers on thermal and nanoparticle properties [35]. Three different molecular weights of PCL were used, namely 570, 1140, and 2280 Da. Thermal analysis showed that both PCL and TPGS affected the crystallization and melting of the other. The DSC thermogram of TPGS alone showed an endothermic peak at 35.7°C, which is close to what is reported herein and within the melting temperature reported in the literature (37–41° C). However, one distinct endothermic peak at lower temperature (21.4 °C for TPGS_1000_-*b*-PCL_570_) and bimodal melting peaks at higher temperatures were observed for TPGS_1000_-*b*-PCL_1140_ (32.1 °C and 39.5 °C) and TPGS_1000_-*b*-PCL_2280_ (41.7 °C and 48.2 °C) due to the formation of two separate microdomains of TPGS and PCL [35]. Here, all the endothermic peaks we observed for TPGS-*b*-PCL were at temperatures higher (51–61 °C) than that obtained for TPGS alone because the PCL molecular weights used were all higher (PCL MW ≥ 2000 Da) than those used in the study by Suksiriworapong et al. [35]. Nonetheless, the DSC thermograms observed here for TPGS_2000_, TPGS_3500_, and TPGS_5000_ and their PCL copolymers showed a trend similar to that reported by Suksiriworapong et al. [35]. Specifically, TPGS-*b*-PCL copolymers had bimodal melting peaks at temperatures that were generally lower than those obtained for the corresponding TPGS. Moreover, the melting peaks became sharper and appeared at higher temperatures when the molecular weight of PCL increased in the TPGS-*b*-PCL copolymers, as depicted in Figure 5. 

This phenomenon has also been previously reported with the closely related PEO-*b*-PCL copolymers, which only lack the α-tocopheryl succinate moiety that is attached to one side of the PEO. For instance, Bogdanov et al. found that PCL blocks crystallize first, which determines the total copolymer structure and leads to imperfect crystallization of the PEO blocks [48]. This was supported by other studies that revealed crystallizability of each block was determined by the relative block length in PEO-*b*-PCL copolymers [37,49,50]. Furthermore, Sun et al. studied the crystallization process of a series of PEO-*b*-PCL copolymers with asymmetric block compositions [42]. The crystallization behaviors of PEO-*b*-PCLs were studied using differential DSC and temperature-dependent XRD. They observed that PEO-*b*-PCL copolymers undergo microphase separation because the PCL and PEO blocks tend to crystallize separately. Moreover, the DSC thermogram of PEO_5000_-*b*-PCL_2900_ showed a bimodal melting peak at 51.0° and 54.9 °C, whereas the copolymer with the higher molecular weight PCL (i.e., PEO_5000_-*b*-PCL_9200_) showed a sharper melting peak at a higher temperature (56.7 °C) [42]. 

### 3.2. Preparation and Characterization of Drug-Free and PAX-Loaded TPGS-PCL Nanocarriers

**Figure 6:**
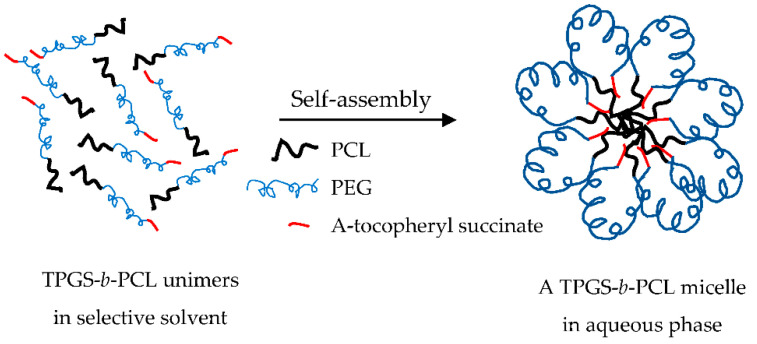
Proposed model for TPGS-*b*-PCL micelle formation in water.

At the beginning, several water-miscible organic solvents were used (including acetonitrile, tetrahydrofuran, and acetone) to prepare micelles using the cosolvent evaporation method. Each organic solvent was tried in different ratio combinations with the aqueous phase, i.e., deionized water (1:1, 1:2, and 1:6). Unfortunately, none of the drug-free or paclitaxel-loaded TPGS_1000_-*b*-PCL copolymers formed micelles with an acceptable yield or size even when other methods of preparation were used, e.g., film hydration method. On the other hand, all the copolymers prepared from TPGS with larger molecular weight PEG (2000–5000 Da) formed micelles with mean diameters < 200 nm and with good yield (minimal or no precipitation). It seems that a PEG chain equal to or larger than 2000 Da was needed to provide the necessary folding to bring the tocopheryl succinate moiety (hydrophobic) in proximity to PCL, the core-forming block, during micelle formation in water. It is believed that this is the thermodynamically favorable orientation for TPGS-*b*-PCL copolymers during the micellization process in aqueous phase (Figure 6), which is similar to the one reported for flower-like micelles formed from A-B-A triblock copolymers, where B is the hydrophilic block (e.g., Pluronic-R) [51]. However, this is only a hypothetical model that needs to be verified. Further studies are needed to investigate the conformation of these micelles in water (e.g., by using ^1^H NMR in D_2_O) [52,53] and to examine the morphology of the self-assembled structures in water (e.g., by using cryogenic TEM or atomic force microscopy) [54,55,56]. 

#### 3.2.1. Size, Polydispersity, and CMC

Based on our previous experience with PEO-*b*-PCL block copolymers, the cosolvent evaporation method is one of the best approaches to prepare monodisperse and stable micelles [57]. We have successfully used this method to load different drugs including cyclosporine A [57], valspodar [11], tacrolimus [58], nimodipine [59], and paclitaxel [60]. Compared to dialysis, which is another commonly used method, cosolvent evaporation method offers several advantages, including greater feasibility of scale up and less chance of drug loss during the encapsulation process [57,61].

Drug-free TPGS-*b*-PCL micelles were successfully prepared using the cosolvent evaporation method. The mean diameters ranged from 70 to 170 nm with a unimodal particle distribution (Figure S6, Table 1). Drug loading did not seem to have an influence on the size of the micelles (Table 2). The difference was statistically significant only in TPGS_2000_-PCL_2000_, TPGS_2000_-PCL_6000_, and TPGS_3500_-PCL_10500_ micelles (*p* < 0.05; paired Student’s *t*-test). In fact, all these micelles showed secondary peaks in the DLS, which may explain the variations in their diameters (Figure S7). The drug-free TPGS-*b*-PCL micelles had a relatively low polydispersity (≤0.27) except for TPGS_2000_-*b*-PCL_2000_, TPGS_2000_-*b*-PCL_4000_, and TPGS_3500_-*b*-PCL_3500_, which had intermediate polydispersity values ranging from 0.35 to 0.54 (Table 1). TPGS_2000_-*b*-PCL copolymers also had the highest polydispersity values among all the prepared PAX-loaded nanocarriers (Table 2). In fact, the size distributions of both drug-free and PAX-loaded TPGS_2000_-*b*-PCL nanocarriers showed bimodal size distribution (Figure S7), which may suggest either nanoparticle aggregation or formation of a mixed population, e.g., micelles and polymersomes. However, further studies (e.g., atomic force microscopy and cryogenic TEM) are needed to verify the possibility of presence of mixed morphologies [54,56,62]. 

The CMC of prepared TPGS-*b*-PCL micelles was determined by DLS, as previously reported [28,59]. Although fluorescent spectroscopy techniques are commonly used for determination of CMC, the application of DLS is expected to reduce the chance of error in CMC measurement. Since the measurement of CMC using fluorescent probes, such as pyrene, heavily depends on the partition equilibrium coefficient (kV) of the fluorescent probe to the core of micelle solution, the physical state of the micellar core may affect this partition and thus influence the measured CMC values [56]. 

The calculated CMC values of TPGS-*b*-PCL micelles were all in the micromolar range (Table 1; Figure S8). Generally, for polymeric micelles, the larger the molecular weight of the hydrophobic block used, the lower the CMC value obtained. Here, although the CMC data followed the expected trend when each set of TPGS-*b*-PCL micelles was compared to the others, most of the differences were not statistically significant (*p* > 0.05; ANOVA followed by Tukey–Kramer post hoc analysis) (Table 1). The CMC values of TPGS-*b*-PCL copolymers were 4.5- to 22.7-fold lower than the respective (unmodified) TPGS, depending on the molecular weight of both TPGS and PCL (Table 1). Indeed, as expected, the higher the molecular weight of PCL in each TPGS derivative, the lower the CMC value obtained. 

#### 3.2.2. Morphology

**Figure 7:**
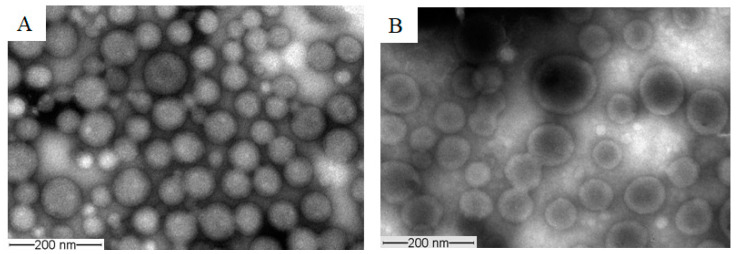
Representative TEM images obtained from unloaded TPGS_5000_-*b*-PCL_15000_ (**A**) and PAX-loaded TPGS_5000_-*b*-PCL_15000_ nanocarriers (at 1:10 drug-to-polymer ratio) (**B**) using a JEOL JEM-1400 transmission electron microscope (JAPAN) operating at an acceleration voltage of 80 kV.

The morphology of the prepared unloaded and PAX-loaded micelles was studied by TEM, which revealed that the micelles had spherical shapes (Figure 7). The diameters of unloaded and PAX-loaded micelles were found to be in agreement with the DLS results.

#### 3.2.3. Encapsulation Efficiency and Drug Loading

Nine polymeric micellar formulations of paclitaxel were prepared (Table 2). The drug was loaded at different levels in each formulation. The highest drug loading % was 1.05%, which was achieved with TPGS_3500_-*b*-PCL_7000_ at a drug:polymer ratio of 1:10 (Table 2). The values were lower than those reported with TPGS-*b*-PCL_10500_ and TPGS-*b*-PCL_30000_ nanoparticles (1.2–6.0%) [22,23]. The lower PAX loading in polymeric micelles is likely because PAX is a bulky molecule (MW = 853.9 Da), and since it is also very hydrophobic, it needs to be loaded inside the hydrophobic core of the micelles. In contrast, PAX can be physically embedded into the polymer matrix or adsorbed onto the surface of the polymeric nanoparticles, which may offer a higher capacity for drug loading. The PAX-loaded micelles we reported here had drug % encapsulation efficiencies ranging from 6.7 to 17.7%. The highest PAX % encapsulation efficiency was obtained with TPGS_2000_-*b*-PCL_4000_ (17.24%), TPGS_3500_-*b*-PCL_7000_ (17.28%), and TPGS_5000_-*b*-PCL_15000_ (17.68%) (Table 2). This translates to an increase in aqueous solubility of PAX from 0.3 µg/mL [15] to 88.4 µg/mL, i.e., approximately a 300-fold increase in its aqueous solubility. This aqueous solubility value is about 120% higher than the value reported for PAX-loaded PEO_5000_-*b*-PCL_5000_ micelles (~40 µg/mL) when different drug:polymer ratios were evaluated (1:10–1:50) [60]. The % encapsulation efficiency of PAX did not seem to be influenced by the variations in the molecular weight of TPGS or PCL. 

### 3.3. In Vitro Release of PAX from the Nanocarriers

**Figure 8:**
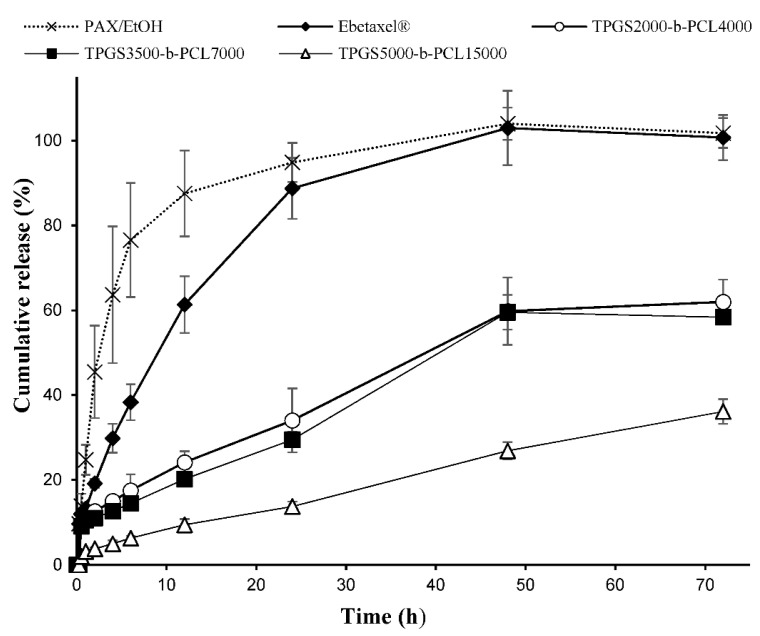
Release profiles of PAX from the ethanolic solution (PAX/EtOH), commercial formulation (Ebetaxel^®^), and the polymeric micellar formulations (TPGS_2000_-*b*-PCL_4000_, TPGS_3500_-*b*-PCL_7000_, and TPGS_5000_-*b*-PCL_15000_, prepared at 1:15 drug:polymer ratio) in phosphate-buffered saline (PBS; pH 7.4) containing 0.5% *w*/*v* polysorbate 80 at 37 °C and 100 rpm. Each data point represents the mean ± SD (n = 3).

The polymeric micellar formulation that showed the highest % drug encapsulation efficiency was chosen from each TPGS group. The in vitro release profiles of PAX from TPGS_2000_-*b*-PCL_4000_, TPGS_3500_-*b*-PCL_7000_, and TPGS_5000_-*b*-PCL_15000_ are represented in Figure 8. All three micellar formulations exhibited a sustained release of PAX. TPGS_2000_-*b*-PCL_4000_ and TPGS_3500_-*b*-PCL_7000_ had comparable PAX release profiles, showing only around 20% drug release within the first 12 h and approximately 60% release within 72 h. TPGS_5000_-*b*-PCL_15000_, on the other hand, seemed to exert more control over the release of the encapsulated drug, showing less than 10% release within the first 12 h, and only 36% release within 72 h. This contrasts with the profiles obtained for the controls, i.e., ethanolic solutions of PAX and Ebetaxel^®^, where there was around 88% and 61% cumulative release of the drug within the first 12 h of incubation. Moreover, the drug was entirely released from both controls within 48 h of incubation, which provides evidence that the experimental setup was valid, i.e., the sink condition was met and the dialysis membrane did not act as a barrier to drug release. The results also showed that TPGS-*b*-PCL nanocarriers were able to control the release of the drug.

The release profiles of PAX from TPGS_2000_-*b*-PCL_4000_ and TPGS_3500_-*b*-PCL_7000_ were found to be comparable to that reported for the PAX-loaded PEO_5000_-*b*-PCL_5000_ micelles at a similar conditions, where around 50% of PAX was released within 72 h [60]. However, the drug release pattern obtained with TPGS_5000_-*b*-PCL_15000_ more closely resembled the ones reported for PAX loaded into TPGS_1000_-*b*-PCL_10500_, PEO_5000_-*b*-PCL_9500_, and TPGS_1000_-*b*-PCL_30000_ nanoparticles under similar conditions [22,23]. 

The different release profiles of PAX from the ethanolic solution, Ebetaxel^®^, and the three TPGS-*b*-PCL micellar formulations were analyzed by measuring the difference (*f*_1_) and similarity (*f*_2_) factors (Table 3). The results showed that the release of PAX from the commercial formulation, i.e., Ebetaxel^®^, was different from that of the free drug (PAX ethanolic solution), where the calculated *f*_1_ and *f*_2_ values were 23.72 and 32.75, respectively. Indeed, the release of PAX in Ebetaxel^®^ was relatively slower compared to the PAX/EtOH, especially in the first 12 h. Ebetaxel^®^, like the brand product Taxol, is known to form micelles when diluted in water due to the presence of polyethoxylated castor oil i.e., Kolliphor^®^ EL. It seemed that the micelles formed from Kolliphor^®^ EL had an influence over the release of PAX in a similar manner to what we previously reported with cyclosporine A in its commercial formulation (Sandimmune^®^), which also contain Kolliphor^®^ EL [63].

The calculated *f*_1_ and *f*_2_ values showed that the release profiles of both PAX/EtOH and Ebetaxel^®^ were different from all the three TPGS-*b*-PCL micellar formulations. Moreover, PAX had a similar release profile from TPGS_2000_-*b*-PCL_4000_ and TPGS_3500_-*b*-PCL_7000_, suggesting similar release kinetics. However, the drug release from TPGS_5000_-*b*-PCL_15000_ was shown to be different than that from all the others. Overall, the release study demonstrated the capability of TPGS-*b*-PCL micelles to control the release of PAX compared to the commercially available formulation.

## 4. Conclusions

Although none of the TPGS-*b*-PCL copolymers (with PEG MW of 1000) were able to form micelles, all copolymers that consisted TPGS with high-molecular-weight PEG (≥ 2000 Da) had formed micelles. The mean diameters of the prepared self-assembled structures were in the nano range (≤ 200 nm). Moreover, TPGS-*b*-PCL micelles significantly increased the aqueous solubility of PAX from 0.3 µg/mL to 88 µg/mL. The developed micelles were also shown to significantly control the in vitro release of PAX. Compared to a 100% drug release obtained with the commercially available formulation within 72 h, the TPGS_5000_-*b*-PCL_15000_ micellar formulation showed only 36% cumulative drug release over the same period with no burst release. Our results indicate a great potential for TPGS-*b*-PCL micelles to serve as efficient solubilizing and delivery system for PAX and potentially for other hydrophobic drugs.

## Figures and Tables

**Table 1:** Characteristics of the synthesized TPGS-*b*-PCL copolymers and their self-assembled nanocarriers.

Block Copolymer ^a^	Theoretical Molecular Weight (g/mol)	*M*_n_ (g/mol) ^b^	*M*_n_ (g/mol) ^c^	Đ ^d^	Diameter ^e^ (nm)	Polydispersity ^e^	CAC ^f^ (µM)
TPGS_1000_	1513	-	-	-	-	-	132.2 ^g^
TPGS_1000_-*b*-PCL_2050_^§^	3560	3560	5330	1.06	-	-	-
TPGS_1000_-*b*-PCL_4200_	5500	5700	9000	1.29	-	-	-
TPGS_1000_-*b*-PCL_8000_	9500	9500	15,200	1.27	-	-	-
TPGS_1000_-*b*-PCL_15200_	17,500	16,700	23,100	1.17	-	-	-
TPGS_1000_-*b*-PCL_20800_	21,500	22,300	35,100	1.10	-	-	-
TPGS_1000_-*b*-PCL_25100_	26,600	26,600	38,950	1.11	-	-	-
TPGS_1000_-*b*-PCL_30400_	31,500	31,900	43,250	1.14	-	-	-
TPGS_2000_	2513	-	-	-	-	-	186.2 ± 16.2
TPGS_2000_-*b*-PCL_2000_	4500	4550	5950	1.25	70.5 ± 6.2	0.54 ± 0.30	41.00 ± 3.06 *
TPGS_2000_-*b*-PCL_4000_	6500	6400	8350	1.55	86.5 ± 3.9	0.45 ± 0.04	25.16 ± 3.10 *
TPGS_2000_-*b*-PCL_6000_	8500	8800	10,500	1.71	170.1 ± 9.4	0.21 ± 0.02	18.06 ± 0.20 *^#^
TPGS_3500_	4013	-	-	-	-	-	165.7 ± 15.0
TPGS_3500_-*b*-PCL_3500_	7500	7100	4600	1.81	89.2 ± 4.1	0.35 ± 0.06	24.00 ± 1.81 *
TPGS_3500_-*b*-PCL_7000_	11,000	10,300	6400	1.62	61.3 ± 1.7	0.27 ± 0.02	15.88 ± 2.41 *
TPGS_3500_-*b*-PCL_10500_	14,500	13,900	10,300	1.62	81.7 ± 3.6	0.23 ± 0.03	8.38 ± 0.88 *
TPGS_5000_	5513	-	-	-	-	-	123.6 ± 6.8
TPGS_5000_-*b*-PCL_5000_	10,500	10,300	4600	1.90	81.1 ± 2.8	0.27 ± 0.06	12.30 ± 0.82 *
TPGS_5000_-*b*-PCL_10000_	15,500	15,600	8100	1.53	75.0 ± 4.6	0.19 ± 0.05	7.29 ± 0.89 *
TPGS_5000_-*b*-PCL_15000_	20,500	20,550	12,100	1.70	77.0 ± 4.6	0.21 ± 0.03	5.44 ± 0.36 *

^a^ The number shown as a subscript indicates the molecular weight of each block determined by ^1^H NMR. ^b^ Number-average molecular weight measured by ^1^H NMR. ^c^ Number-average molecular weight measured by GPC using PS standards. ^d^ Dispersity (Đ) determined by GPC. ^e^ Diameter and polydispersity of the nanocarriers estimated by the DLS technique. ^f^ Critical association concentration (CAC) measured by DLS. ^g^ reported in the literature (0.02% *w*/*v*). ^§^ TPGS_1000_-*b*-PCL copolymers did not form micelles. * Significantly different form the respective unmodified TPGS (*p* < 0.05; one-way ANOVA followed by Tukey–Kramer post hoc test). ^#^ Significantly different from TPGS_2000_-*b*-PCL_2000_ (*p* < 0.05; one-way ANOVA followed by Tukey–Kramer post hoc test).

**Table 2:** Characteristics of the prepared PAX-loaded nanocarriers.

Block Copolymer	Drug: Polymer Ratio (*w*/*w*)	Drug Loading (% *w*/*w*) ^a^	Encapsulation Efficiency (%) ^a^	Diameter (nm) ^b^	Polydispersity ^b^
**TPGS_2000_-*b*-PCL_2000_**	1:10	0.66 ± 0.01	6.70 ± 0.13	195.1 ± 31.8 *	0.52 ± 0.18
	1:20	0.35 ± 0.01	7.10 ± 0.08	131.1 ± 69.7	0.46 ± 0.13
	1:30	0.32 ± 0.02	8.04 ± 0.39	155.1 ± 31.3	0.53 ± 0.10
**TPGS_2000_-*b*-PCL_4000_**	1:10	0.85 ± 0.01	8.60 ± 0.08	93.5 ± 11.4	0.50 ± 0.13
	1:20	0.60 ± 0.02	11.98 ± 0.44	77.2 ± 11.4	0.39 ± 0.06
	1:30	0.57 ± 0.01	17.24 ± 0.14	77.6 ± 6.5	0.40 ± 0.06
**TPGS_2000_-*b*-PCL_6000_**	1:10	0.75 ± 0.05	7.54 ± 0.54	209.4 ± 5.1 *	0.25 ± 0.03
	1:20	0.52 ± 0.02	10.54 ± 0.45	242.7 ± 7.5	0.43 ± 0.10
	1:30	0.27 ± 0.01	8.04 ± 0.39	199.2 ± 45.3	0.46 ± 0.10
**TPGS_3500_-*b*-PCL_3500_**	1:10	1.00 ± 0.05	10.07 ± 0.62	81.9 ± 7.8	0.33 ± 0.03
	1:20	0.63 ± 0.03	12.67 ± 0.54	85.1 ± 6.8	0.34 ± 0.11
	1:30	0.47 ± 0.02	13.24 ± 0.36	74.4 ± 1.3	0.30 ± 0.03
**TPGS_3500_-*b*-PCL_7000_**	1:10	1.05 ± 0.03	10.59 ± 0.26	74.8 ± 9.5	0.33 ± 0.11
	1:20	0.63 ± 0.02	12.58 ± 0.47	60.6 ± 1.0	0.31 ± 0.03
	1:30	0.60 ± 0.02	17.28 ± 0.53	67.2 ± 2.0	0.21 ± 0.01
**TPGS_3500_-*b*-PCL_10500_**	1:10	0.83 ± 0.02	8.42 ± 0.19	91.4 ± 4.1 *	0.32 ± 0.01
	1:20	0.58 ± 0.01	11.68 ± 0.19	80.1 ± 0.7	0.24 ± 0.02
	1:30	0.57 ± 0.03	17.10 ± 1.00	94.0 ± 1.1	0.25 ± 0.01
**TPGS_5000_-*b*-PCL_5000_**	1:10	0.85 ± 0.05	8.55 ± 0.51	83.7 ± 3.0	0.31 ± 0.01
	1:20	0.59 ± 0.04	11.58 ± 0.51	92.7 ± 1.0	0.21 ± 0.01
	1:30	0.51 ± 0.02	14.32 ± 0.46	96.1 ± 1.3	0.16 ± 0.03
**TPGS_5000_-*b*-PCL_10000_**	1:10	0.84 ± 0.01	8.45 ± 0.06	76.6 ± 0.6	0.21 ± 0.04
	1:20	0.63 ± 0.03	12.76 ± 0.57	79.7 ± 3.8	0.20 ± 0.02
	1:30	0.52 ± 0.02	15.72 ± 0.60	79.1 ± 2.3	0.19 ± 0.03
**TPGS_5000_-*b*-PCL_15000_**	1:10	0.80 ± 0.02	8.06 ± 0.19	76.4 ± 2.9	0.22 ± 0.02
	1:20	0.62 ± 0.02	12.56 ± 0.49	85.0 ± 0.7	0.16 ± 0.02
	1:30	0.59 ± 0.03	17.68 ± 0.30	93.2 ± 1.0	0.18 ± 0.05

^a^ The amount of drug in each nanocarrier was determined using an HPLC assay. ^b^ Average diameter (Z_ave_) and polydispersity were estimated using the DLS technique. Data are presented as mean ± SD (n = 3). * Significantly different from the unloaded counterpart presented in Table 1 (*p* < 0.05; Paired Student’s *t*-test).

**Table 3:** Calculated difference factors (*f*_1_) and similarity factors (*f*_2_) for PAX release profiles from ethanolic solution (PAX/EtOH), commercial formulation (Ebetaxel^®^), and TPGS-*b*-PCL micellar formulations. The profiles are considered similar if *f*_1_ ≤ 15 and *f*_2_ ≥ 50.

Solution/Formulation	Difference Factor (*f*1)	Similarity Factor (*f*2)
PAX/EtOH vs. Ebetaxel^®^	23.72	32.75
Ebetaxel^®^ vs. TPGS2000-*b*-PCL4000	48.45	24.28
Ebetaxel^®^ vs. TPGS3500-*b*-PCL7000	52.62	22.66
Ebetaxel^®^ vs. TPGS5000-*b*-PCL15000	77.64	14.81
TPGS2000-*b*-PCL4000 vs. TPGS3500-*b*-PCL7000 *	8.09	75.75
TPGS2000-*b*-PCL4000 vs. TPGS5000-*b*-PCL15000	56.62	36.38
TPGS3500-*b*-PCL7000 vs. TPGS5000-*b*-PCL15000	52.80	38.93

* similar release profile.

## Data Availability

All data presented in this study are available upon request from the corresponding author..

## Supplementary Materials

The following are available online, Figure S1: Representative ^1^H NMR spectra of TPGS_2000_-*b*-PCL_6000_ (A), TPGS_3500_-*b*-PCL_10500_ (B), and TPGS_5000_-*b*-PCL_15000_ (C); Figure S2: GPC chromatograms of TPGS_1000_ and TPGS_1000_-*b*-PCL_20800_, TPGS_1000_-*b*-PCL_25100_, and TPGS_1000_-*b*-PCL_31500_ copolymers; Figure S3: FTIR spectra of TPGS_1000_, TPGS_2000_, TPGS_3500_ and their corresponding TPGS-*b*-PCL copolymers; Figure S4: XRD spectra TPGS_1000_, TPGS_2000_, TPGS_3500_ and their corresponding TPGS-*b*-PCL copolymers; Figure S5: DSC thermograms of TPGS_1000_-*b*-PCL_20800_, TPGS_1000_-*b*-PCL_25100_, and TPGS_1000_-*b*-PCL_31500_ copolymers; Figure S6: Representative size distribution profiles of (A) unloaded TPGS_5000_-*b*-PCL_15000_ nanocarriers and (B) PAX-loaded TPGS_5000_-*b*-PCL_15000_ nanocarriers obtained by dynamic light scattering (Zetasizer Nano ZS, Malvern Instrument Ltd., UK). The concentration of block copolymers was 10 mg/mL; Figure S7: Representative size distribution profiles of (A) unloaded TPGS_2000_-*b*-PCL_4000_ nanocarriers and (B) PAX-loaded TPGS_2000_-*b*-PCL_4000_ nanocarriers obtained by dynamic light scattering (Zetasizer Nano ZS, Malvern Instrument Ltd., UK) showing bimodal size population. The concentration of block copolymers was 10 mg/mL; Figure S8: Representative plots of scattered intensity (kcps) as a function of TPGS or TPGS-*b*-PCL concentration (µM). The CMC value was taken from the intersection of the best fit lines.
