# Supplementary material for: Design and Development of D‒α‒Tocopheryl Polyethylene Glycol Succinate‒block‒Poly(ε-Caprolactone) (TPGS−b−PCL) Nanocarriers for Solubilization and Controlled Release of Paclitaxel
Source: Molecules. 2021 May 4;26(9):2690. doi: 10.3390/molecules26092690 (PMC8125698; doi:10.3390/molecules26092690)
Supplement: Supplementary file 1 [file molecules-26-02690-s001.zip › molecules-1175098-supplementary.pdf]

Supplementary Materials: Design and development of D–α–Tocopheryl Polyethylene Glycol Succinate–*block*–Poly(ε-caprolactone) (TPGS−*b*−PCL) Nanocarriers for Solubilization and Controlled Release of Paclitaxel

Osman Yusuf, Raisuddin Ali, Abdullah H. Alomrani, Aws Alshamsan, Abdullah K. Alshememry, Abdulaziz M. Almalik, Afsaneh Lavasanifar, and Ziyad Binkhathlan *

(A)

(B)

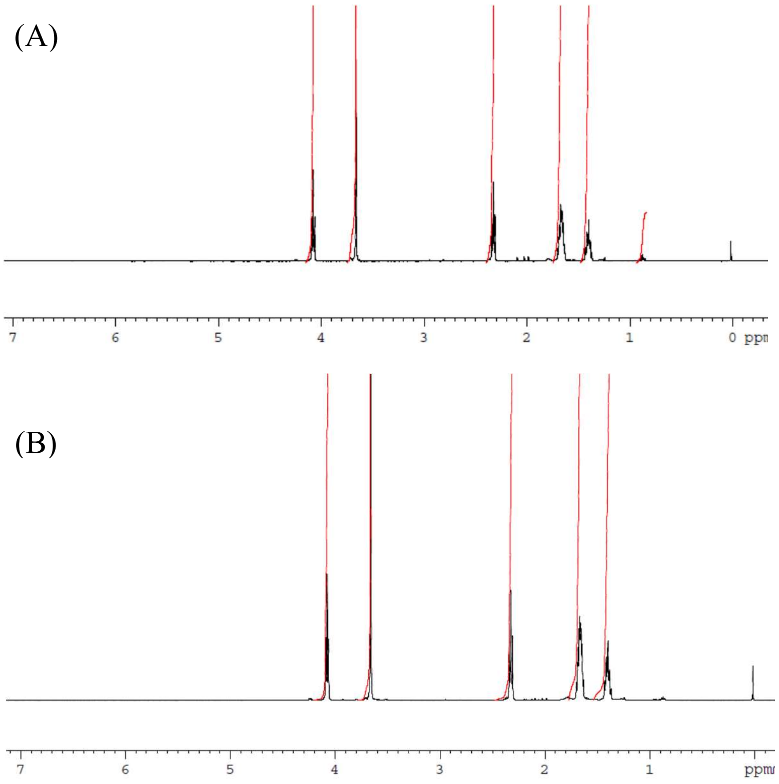

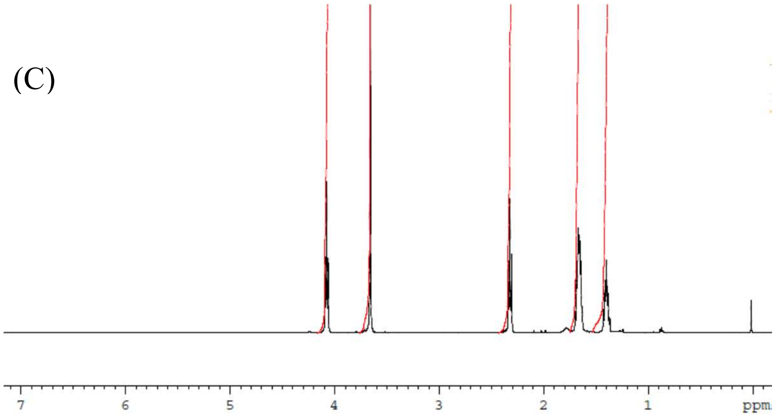

Figure S1. Representative ^{1}H NMR spectra of $TPGS_{2000}$-*b*-PCL_{6000} (A), $TPGS_{3500}$-*b*-PCL_{10500} (B), and $TPGS_{5000}$-*b*-PCL_{15000} (C).

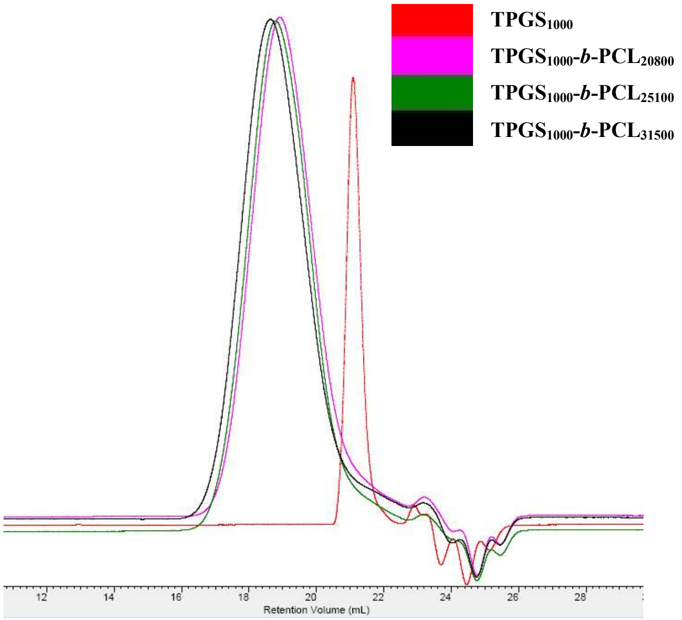

Figure S2. GPC chromatograms of $TPGS_{1000}$ and $TPGS_{1000}$-*b*-PCL_{20800}, $TPGS_{1000}$-*b*-PCL_{25100}, and $TPGS_{1000}$-*b*-PCL_{31500} copolymers.

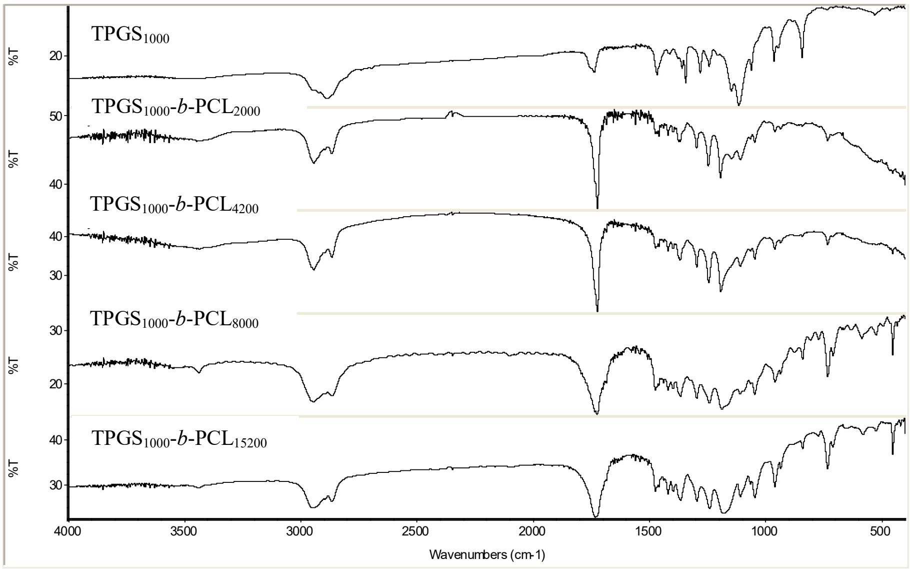
TPGS1000
TPGS1000-b-PCL2000
TPGS1000-b-PCL4200
TPGS1000-b-PCL8000
TPGS1000-b-PCL15200
%T
4000 3500 3000 2500 2000 1500 1000 500
Wavenumbers (cm-1)

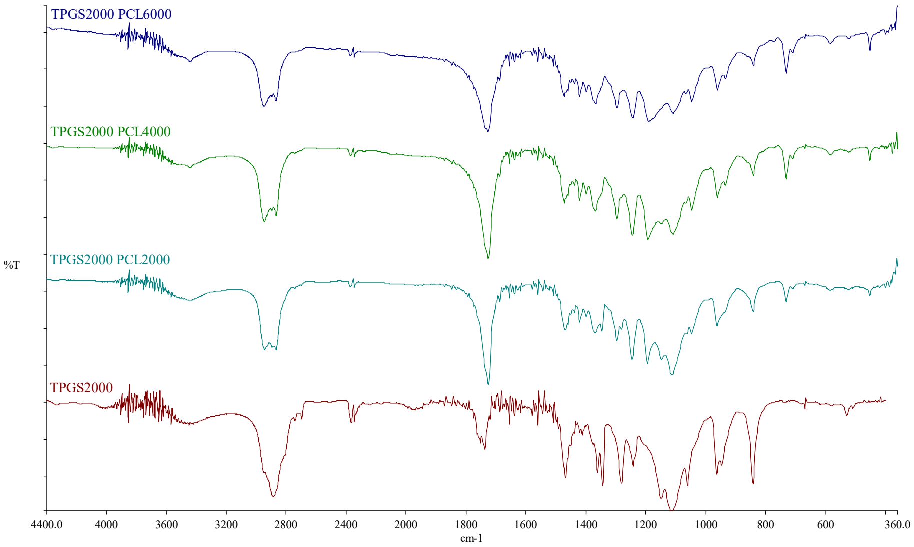
TPGS2000 PCL6000
TPGS2000 PCL4000
TPGS2000 PCL2000
TPGS2000
%T
4400.0 4000 3600 3200 2800 2400 2000 1800 1600 1400 1200 1000 800 600 360.0
cm-1

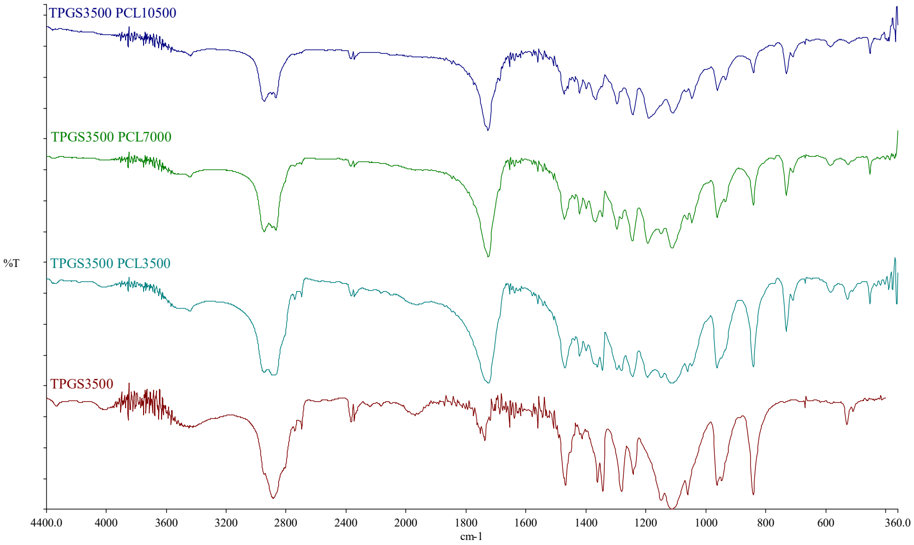
TPGS3500 PCL10500
TPGS3500 PCL7000
TPGS3500 PCL3500
TPGS3500
%T
4400.0 4000 3600 3200 2800 2400 2000 1800 1600 1400 1200 1000 800 600 360.0
cm-1

Figure S3. FTIR spectra of $TPGS_{1000}$, $TPGS_{2000}$, $TPGS_{3500}$ and their corresponding TPGS-*b*-PCL copolymers.

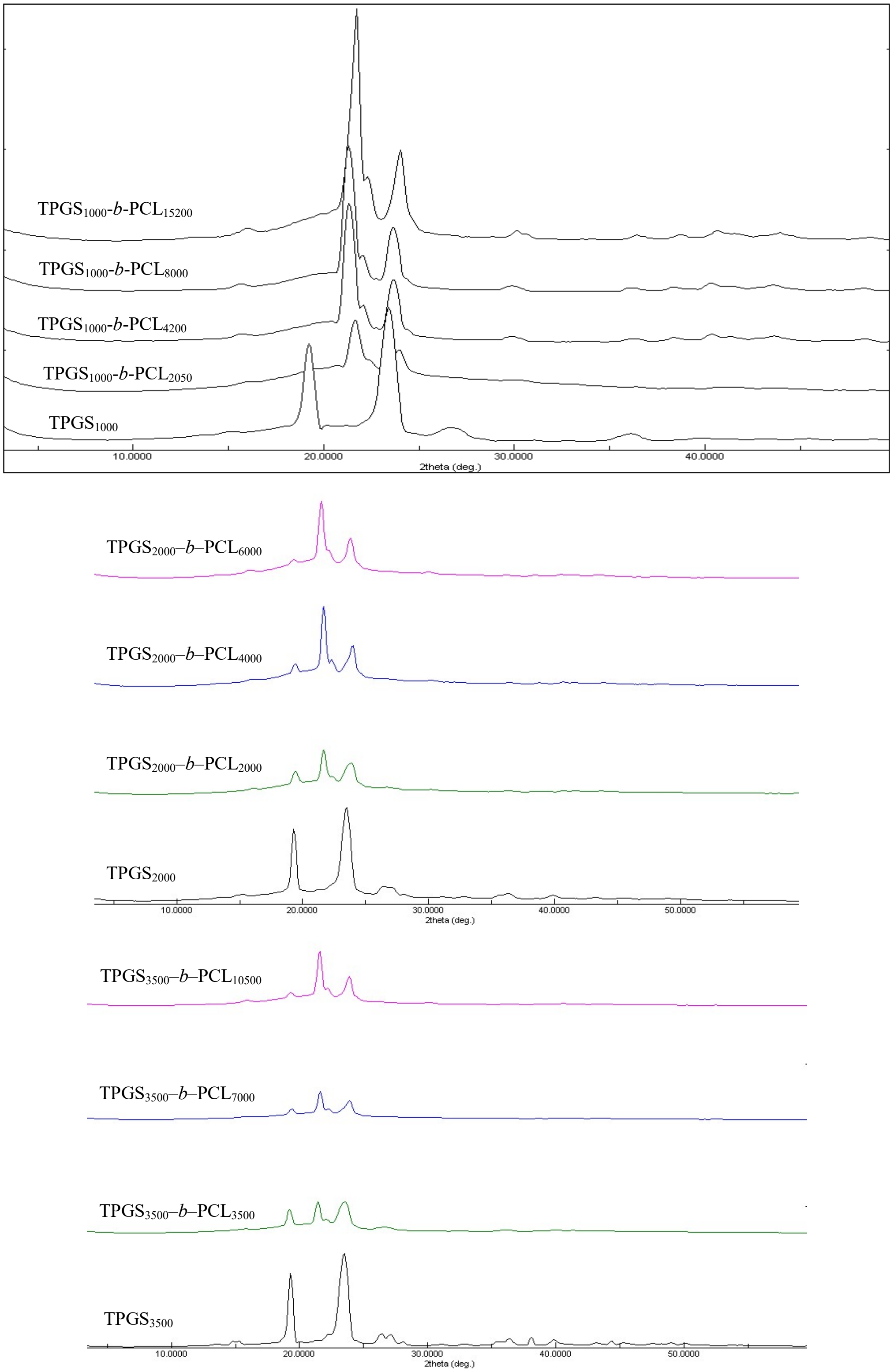

Figure S4. XRD spectra $TPGS_{1000}$, $TPGS_{2000}$, $TPGS_{3500}$ and their corresponding TPGS-*b*-PCL copolymers.

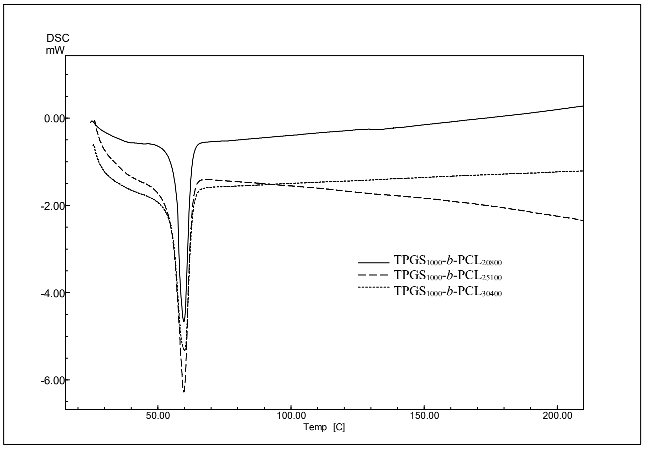

Figure S5. DSC thermograms of $TPGS_{1000}$-*b*-PCL_{20800}, $TPGS_{1000}$-*b*-PCL_{25100}, and $TPGS_{1000}$-*b*-PCL_{31500} copolymers.

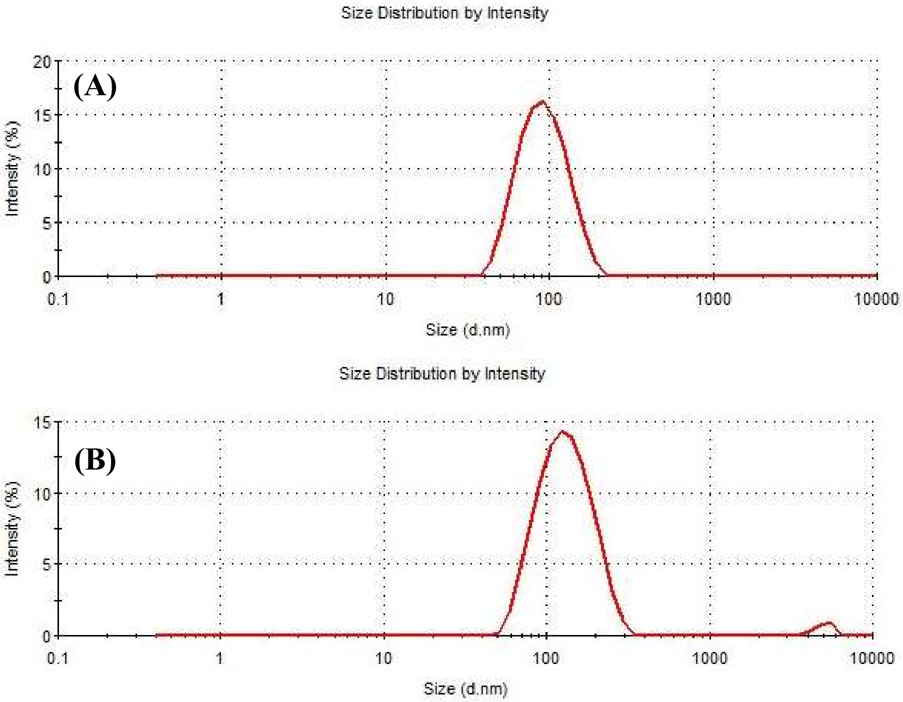

Figure S6. Representative size distribution profiles of (A) unloaded $TPGS_{5000}$-*b*-PCL_{15000} nanocarriers and (B) PAX-loaded $TPGS_{5000}$-*b*-PCL_{15000} nanocarriers obtained by dynamic light

scattering (Zetasizer Nano ZS, Malvern Instrument Ltd., UK). The concentration of block copolymers was 10 mg/mL.

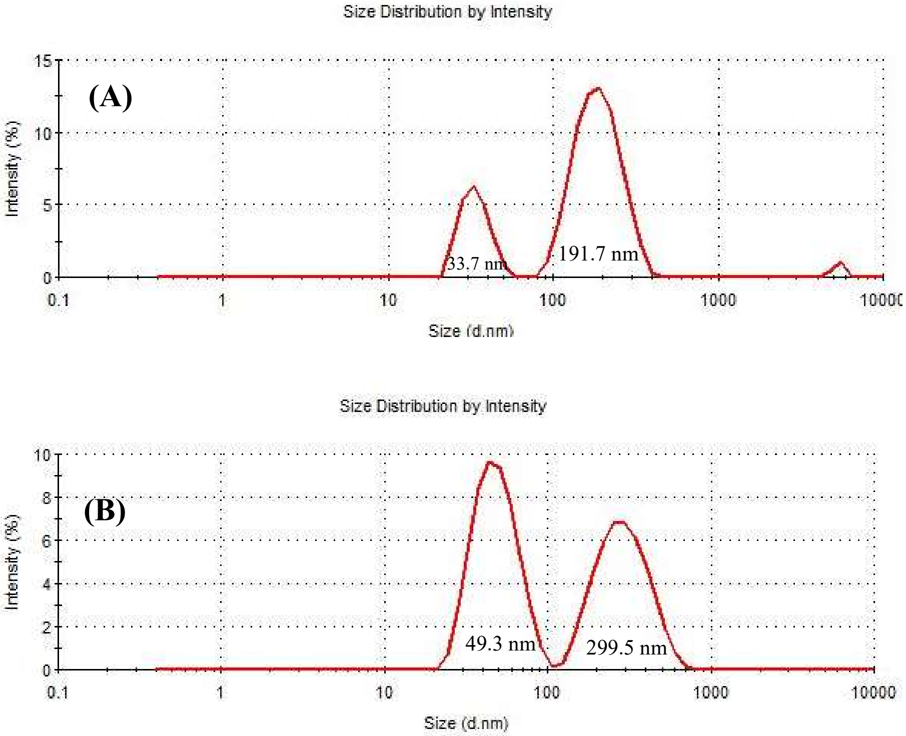

Figure S7. Representative size distribution profiles of (A) unloaded $TPGS_{2000}$-*b*-PCL_{4000} nanocarriers and (B) PAX-loaded $TPGS_{2000}$-*b*-PCL_{4000} nanocarriers obtained by dynamic light scattering (Zetasizer Nano ZS, Malvern Instrument Ltd., UK) showing bimodal size population. The concentration of block copolymers was 10 mg/mL.

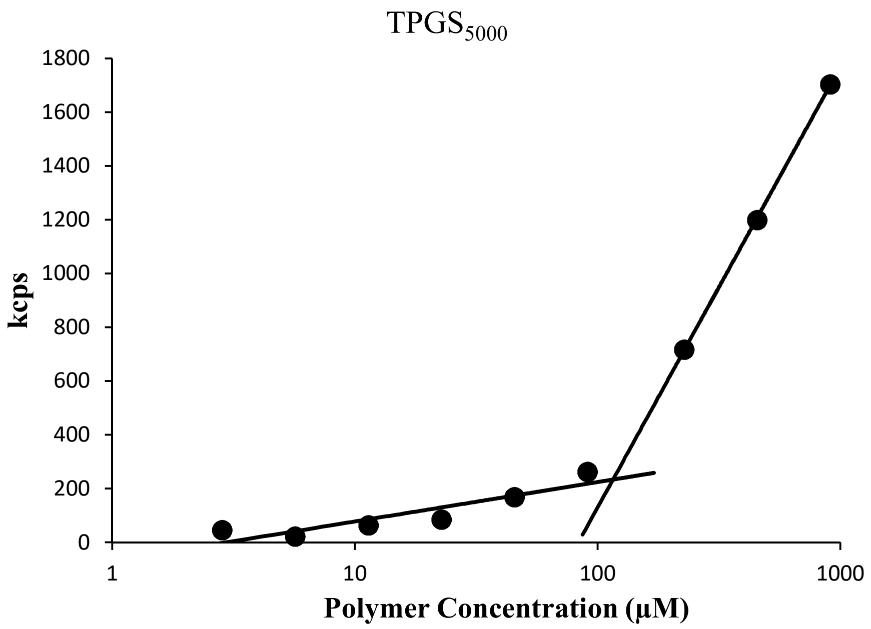
TPGS5000
kcps
1800
1600
1400
1200
1000
800
600
400
200
0
1
10
100
1000
Polymer Concentration (µM)

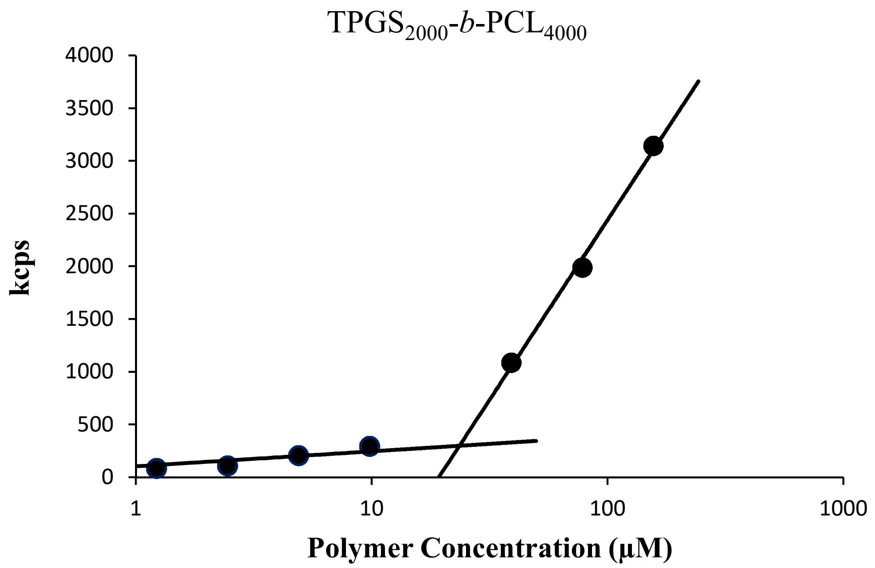
TPGS2000-b-PCL4000
kcps
4000
3500
3000
2500
2000
1500
1000
500
0
1
10
100
1000
Polymer Concentration (µM)

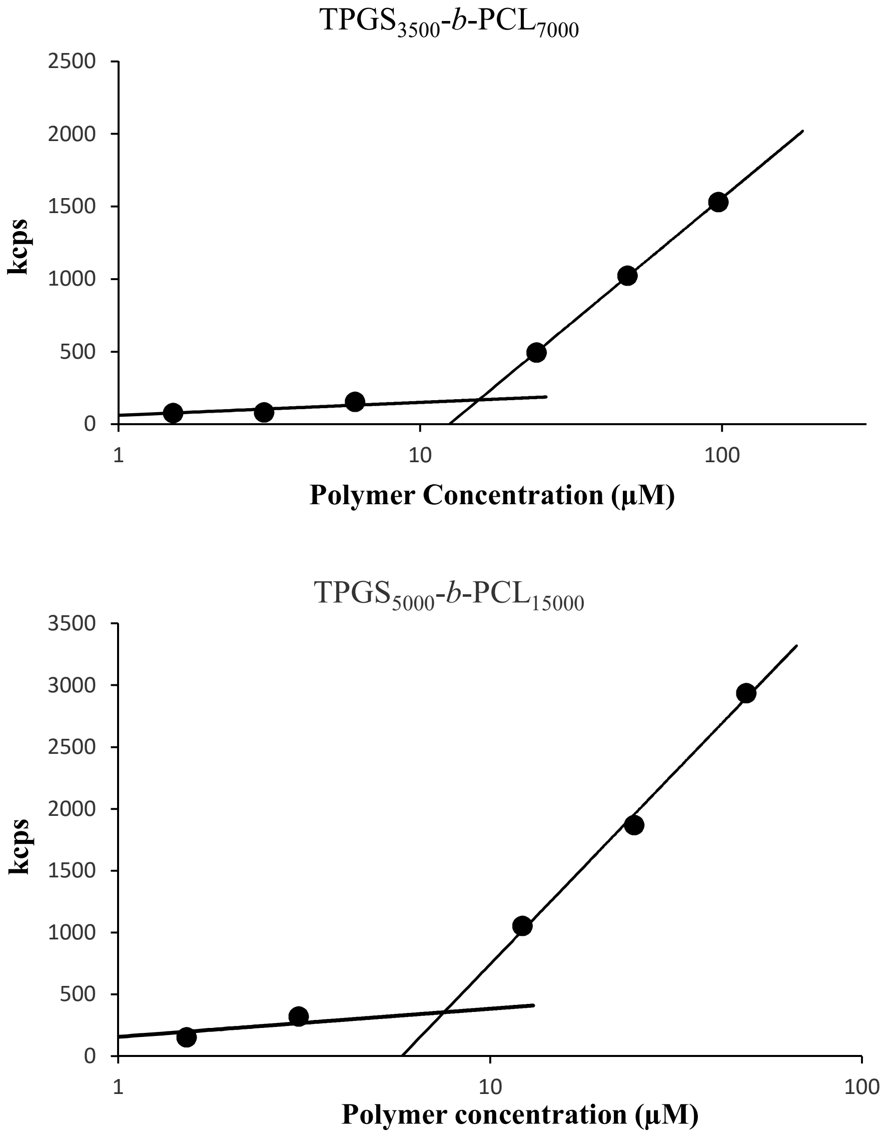

Figure S8. Representative plots of scattered intensity (kcps) as a function of TPGS or TPGS-*b*-PCL concentration (µM). The CMC value was taken from the intersection of the best fit lines.
